# Trust in COVID-19 information sources and vaccination status: Exploring social inequalities and differences within the four United Kingdom nations using a representative survey

**DOI:** 10.1177/13558196241227749

**Published:** 2024-02-05

**Authors:** Valeria Skafida, Elke Heins

**Affiliations:** Social Policy, School of Social and Political Science, 151027 University of Edinburgh, Edinburgh, UK

**Keywords:** national health systems, public health messaging, health information sources

## Abstract

**Objectives:**

To explore how the use of, and trust in, different sources of advice and information on COVID-19 differed across the four UK nations and between different sociodemographic groups and their associations with COVID-19 vaccination status.

**Methods:**

We used a UK-wide representative survey conducted in July 2021, which included data on uptake of COVID-19 vaccination, trust in information sources, use of sources and geographical and sociodemographic variables. We used multivariate logistic regression to identify factors associated with completed or planned COVID-19 vaccination.

**Results:**

Trust in the NHS, followed by trust in scientists, were the strongest predictors of vaccination intention. NHS websites were the most used (56% across the UK); only the Scottish government website had a higher level of reported use (58%). Using either source was associated with a positive vaccination status as were use of the GP and television as sources of advice. Use of social media, family and friends, and ‘none’ of the sources enquired about, were all linked to a lower likelihood of being or intending to get vaccinated. Compared to those in England, respondents in other UK nations were less likely to trust the central UK government for advice on COVID-19. There was considerable variation by age in trust and use of some, but not all, sources of advice, with predicted probabilities ranging from 35% among the youngest age group to 62% among those aged 65 years or older. There were also significant differences by annual household income and by occupational class for trust in government, with higher incomes correlating with greater likelihood of trust.

**Conclusions:**

This study demonstrates high levels of trust in the key sources of public health advice and there was a positive association between using official sources of advice and vaccination intentions, even in the context of overall high vaccination rates. Our findings highlight the need for the UK and devolved governments to value the importance of public trust in the health system and take appropriate measures to avoid undermining such trust.

## Introduction

The roll-out of COVID-19 vaccination in the United Kingdom (UK) can be described as a success. By September 2022, nearly 80% of the UK population had been vaccinated at least once, and over 75% were fully vaccinated at that time.^1^ International evidence highlights the importance of public trust for following public health guidance and vaccine uptake. For example, data from France and Italy indicate that trust in government was linked to increased compliance with rules and restrictions related to COVID-19.^2^ Research from Canada found that people who intended to receive a COVID-19 vaccine described trusted medical experts as positive influences,^3^ while data from New Zealand suggest that communications from scientific experts that addressed prevailing concerns about vaccines were likely to help improve vaccine uptake.^4^

Conversely, belief in conspiracy theories related to COVID-19 predicted significantly lower intention to get vaccinated,^5–7^ as did lack of trust in official information sources^8^ and higher susceptibility to misinformation.^9^ Online surveys across eight high-income countries found large variation in self-reported acceptance of a COVID-19 vaccine; they also showed that vaccination hesitancy was associated with lack of trust in authorities and scientists, as well as with conspiracy beliefs.^10^ Evidence from Portugal found that low confidence in the health service response to the pandemic predicted vaccine hesitancy.^11^

In the UK, the National Health Service (NHS) is held in high regard by the population and rated as the most trusted public service.^12^ This is in contrast to low levels of trust in the national government, which, at 35% in 2022, was lower than the OECD average of 41%. Public health messaging on pandemics is communicated mainly through the NHS but it is not known what role trust in the NHS has had in COVID-19 vaccine uptake and whether and how this may have been influenced by a series of political scandals, such as the apparent breaches of COVID-19 regulations by senior members of the government.^13^ There is also a lack of research on whether and how this varied across sociodemographic groups and the devolved nations of the UK. This study sought to help fill this evidence gap using a cross-sectional, nationally representative survey to explore how the use of and trust in different sources of advice and information on COVID-19 differed (i) across the four UK nations and (ii) between different sociodemographic groups. We further explored the relationship between COVID-19 vaccination status and trust in, and use of, different sources of advice and information on COVID-19.

## Methods

We conducted a survey of adult UK residents (aged 18 years and over) between 16 and 31 July 2021. The survey used an online panel and applied a quota design to ensure that the final sample (*N* = 4428 adults) was representative of the UK population in terms of gender, age, geography and occupational class (for further detail on survey design and sample characteristics please see the Online Supplement).

### Dependent variables

We derived the following dependent variables of interest from survey questions: (i) trust in sources of advice on COVID-19, based on the survey question: *‘There are many people providing information on the COVID-19 pandemic. For each of the following actors, please indicate to what extent you trust or distrust them to provide useful information about the pandemic to you’*, with response categories *‘trust them a lot’* and *‘trust them mostly’* coded as ‘trust’, and categories *‘distrust them mostly’*, *‘distrust them a lot’* and *‘don’t know’* coded as ‘no trust’; (ii) sources used for information on COVID-19, based on the survey question: *‘There are many different sources to get information about COVID-19 from. Which of the following, if any at all, have you personally used to find out more about it? Please select all that apply’*, using all (binary) answer categories (see Table 1 in the Results section); and (iii) vaccination status, which we derived from a survey question on COVID-19 vaccination asking participants if they had been vaccinated yet (and how many doses), and if not, whether they intended to get vaccinated in the future. Since vaccination for all eligible age groups had not been fully rolled out at the time of data collection, we created a binary variable to distinguish between (a) those who had received at least one COVID-19 vaccine and those planning to get one and (b) those who had not yet received a vaccine and were not intending to receive one or who responded ‘prefer not to say’. The latter decision was based on an assumption that vaccine-sceptic participants were more likely to respond this way.

**Table 1.** Weighted descriptive statistics of key variables (*N* = 4428).

	Yes^a^ %	Unweighted *N*
**Trust in source of advice on COVID-19**		
UK government	47.8	2012
Scottish government (*N*:1160)	62.5	722
Welsh government (*N*:670)	64.1	451
Northern Ireland government (*N*:235)	40.3	104
Scientists	77.4	3546
Religious leaders	25.8	1151
The NHS	84.6	3816
Family and friends	78.8	3561
**Sources used for information on COVID-19**		
UK government website	51.7	2145
The Scottish government (N:1160)	58.1	701
Welsh government (N:670)	48.9	351
Northern Ireland government (N:235)	47	121
Devolved government website	21	904
NHS website^b^	56	2460
Print newspaper	18.9	836
Website of a newspaper	20.2	889
Television	50.6	2236
Radio	20.2	905
Social media	19.2	824
Messenger services (e.g. Whatsapp)	4.1	166
Friends and family	28.7	1237
Your GP (General Practitioner)	16.9	709
Leaflets/information brochures sent to you	16.8	823
None of the above	9.6	395
**Vaccination status**		
Received or planning to receive vaccine	92.9	4143
Not received and not planning to receive vaccine (incl. Prefer not to say)	7.1	285

Note. ^a^Yes = 'trust them a lot/mostly’, No = ‘Distrust them a lot/mostly’ and ‘Don’t know’.

^b^can refer to NHS England (https://www.nhs.uk/) or NHS websites in the devolved nations (e.g. Scotland: https://www.nhsinform.scot/).

### Independent variables

We considered the following independent variables: gender (female, male); age (years); self-reported occupational class, using an 8-category scheme where participants selected the occupational category which best described their occupation; and self-reported annual household income (by income band). Because of the large number of missing data for this variable (11%), we included missing income data as a response category in our analyses.

### Analysis

We ran multivariate logistic regression models using Stata 16.1 and calculated ‘Adjusted Predictions at the Means’ using Stata’s margins command. This provided us with predicted probabilities (PP), allowing for comparisons across models.^14^ Predicted probabilities adjust for the effect that other variables in the model have on the outcome. We ran logit models predicting trust in each source of advice on COVID-19 and for different sources of advice used, each controlling for geographic region (England, Northern Ireland, Scotland, Wales) and sociodemographic characteristics. We further ran a series of independent multivariate logit models exploring the relationship between trust in and use of different sources of advice with participant vaccination status. We also ran the latter model disaggregating by UK nation. To explore trust in all sources of advice, we created an additive scale of distrust by pooling all original response items (α = 0.78) and dichotomised the scale to identify the top fifth of the distribution, that is, the most distrusting participants. We recognise that this is a crude index of general trust across sources, and a more granular analysis of underlying trust dimensions (e.g. comparisons of formal vs informal sources) could be explored in future research.

## Results

Table 1 shows descriptive statistics of key variables considered in this study, finding that people are most likely to trust the NHS (85%), followed by trust in family and friends (79%) and in scientists (77%). Religious leaders were trusted least, with only 1 in 4 people trusting them for advice on COVID-19 (26%).

Trust in the UK government was relatively low (48%), compared to trust in the Scottish goverment (63%) and the Welsh government (64%), and lowest for the Northern Ireland government (40%). Among sources used for information on COVID-19, NHS websites were the most used (56% across the UK); only the Scottish government website had a higher level of reported use (58%). The vast majority (93%) of the survey population reported to have had either been vaccinated already, or they were intending to get vaccinated; the remaining 7% had not been and were not planning to get vaccinated, including those preferring not to answer the question.

### Variation by geography and sociodemographic characteristics

Table 2 shows sociodemographic differences in trust in different sources of advice on COVID-19. Compared to England, respondents in the devolved UK nations were less likely to trust the UK government for advice on COVID-19. Differences were particularly pronounced for Scotland compared to England, with predicted probabilities (PP) at 37% and 49% (*p* ≤ 0.001), respectively. There was significant variation in trust in religious leaders, which was lowest in Wales (PP 18%) and highest in Northern Ireland (PP 39%), a difference which was significant at *p* ≤ 0.001.

**Table 2.** Sociodemographic differences in trust in different sources of advice on COVID-19 (predicted probabilities).

	Trust in UK government		Trust in devolved government^a^		Trust scientists		Trust NHS		Trust in family		Trust in religious leaders	
	PP	95%CI	PP	95%CI	PP	95%CI	PP	95%CI	PP	95%CI	PP	95%CI
Nation												
Ref: England	0.49	[0.47,0.52]			0.79	[0.77,0.80]	0.87	[0.85,0.88]	0.79	[0.77,0.81]	0.26	[0.24,0.27]
Ref2^b^: Scotland	0.37***	[0.33,0.40]	0.63	[0.59,0.66]	0.83**	[0.81,0.86]	0.89	[0.86,0.91]	0.83**	[0.80,0.86]	0.24	[0.21,0.27]
Wales	0.42**	[0.38,0.47]	0.64	[0.60,0.68]	0.81	[0.77,0.84]	0.86	[0.83,0.90]	0.80	[0.77,0.84]	0.18***	[0.15,0.21]
Northern Ireland	0.40*	[0.32,0.47]	0.41***	[0.34,0.48]	0.76	[0.69,0.83]	0.87	[0.82,0.92]	0.82	[0.76,0.88]	0.39***	[0.31,0.46]
Gender												
Ref: Female	0.48	[0.45,0.50]	0.61	[0.58,0.65]	0.80	[0.78,0.82]	0.88	[0.86,0.90]	0.81	[0.79,0.82]	0.25	[0.23,0.27]
Male	0.48	[0.45,0.51]	0.57	[0.53,0.61]	0.78	[0.76,0.80]	0.86	[0.84,0.88]	0.78	[0.76,0.80]	0.26	[0.23,0.28]
Age												
Ref: 18–24	0.35	[0.29,0.41]	0.54	[0.43,0.64]	0.72	[0.67,0.77]	0.80	[0.76,0.85]	0.76	[0.71,0.81]	0.24	[0.20,0.29]
25–34	0.38	[0.33,0.43]	0.57	[0.49,0.64]	0.68	[0.63,0.73]	0.73*	[0.68,0.78]	0.73	[0.69,0.78]	0.26	[0.21,0.30]
35–44	0.42	[0.38,0.47]	0.55	[0.49,0.61]	0.74	[0.70,0.78]	0.80	[0.77,0.84]	0.76	[0.72,0.80]	0.26	[0.22,0.30]
45–54	0.47***	[0.42,0.51]	0.60	[0.55,0.66]	0.78*	[0.75,0.82]	0.85	[0.82,0.88]	0.80	[0.77,0.84]	0.24	[0.21,0.28]
55–64	0.53***	[0.49,0.57]	0.63	[0.58,0.68]	0.83***	[0.80,0.86]	0.90***	[0.87,0.92]	0.81	[0.78,0.85]	0.22	[0.18,0.25]
65 or older	0.62***	[0.58,0.66]	0.63	[0.58,0.68]	0.88***	[0.85,0.90]	0.95***	[0.94,0.97]	0.84**	[0.81,0.87]	0.28	[0.25,0.31]
Annual household income												
Ref: Under £19,999	0.45	[0.41,0.48]	0.61	[0.56,0.66]	0.77	[0.74,0.80]	0.84	[0.81,0.87]	0.76	[0.73,0.80]	0.25	[0.22,0.29]
£20,000–£39,999	0.48	[0.45,0.51]	0.62	[0.58,0.67]	0.82*	[0.80,0.85]	0.89**	[0.87,0.91]	0.82*	[0.79,0.84]	0.26	[0.23,0.29]
3.£40,000–£59,999	0.53*	[0.48,0.58]	0.54	[0.47,0.60]	0.81	[0.77,0.85]	0.90**	[0.87,0.93]	0.83*	[0.79,0.87]	0.25	[0.21,0.29]
4.£60,000–£79,999	0.48	[0.41,0.56]	0.56	[0.46,0.66]	0.79	[0.73,0.85]	0.88	[0.83,0.93]	0.80	[0.74,0.86]	0.29	[0.23,0.36]
5.£80,000–£99,999	0.54	[0.43,0.65]	0.55	[0.40,0.71]	0.82	[0.73,0.91]	0.88	[0.81,0.95]	0.83	[0.75,0.91]	0.20	[0.12,0.29]
6.£100,000–£119,999	0.62**	[0.52,0.73]	0.53	[0.35,0.71]	0.83	[0.75,0.91]	0.90	[0.84,0.96]	0.84	[0.76,0.91]	0.35	[0.25,0.46]
Don’t know, prefer not to say	0.42	[0.37,0.48]	0.57	[0.48,0.65]	0.70*	[0.65,0.75]	0.80	[0.75,0.84]	0.71	[0.66,0.76]	0.21	[0.17,0.25]
Occupational class												
Professional or higher technical work	0.48	[0.43,0.53]	0.66**	[0.59,0.72]	0.85***	[0.81,0.88]	0.86	[0.82,0.90]	0.78	[0.74,0.83]	0.32***	[0.27,0.37]
Manager or senior administrator	0.49	[0.44,0.54]	0.63*	[0.57,0.69]	0.84***	[0.80,0.87]	0.88	[0.85,0.91]	0.80	[0.76,0.84]	0.27**	[0.23,0.32]
Junior manager	0.50	[0.45,0.56]	0.64*	[0.57,0.71]	0.80**	[0.76,0.85]	0.90	[0.86,0.93]	0.78	[0.73,0.82]	0.29**	[0.24,0.35]
Non-managerial, non-manual work	0.49	[0.44,0.54]	0.62*	[0.55,0.68]	0.83***	[0.80,0.87]	0.88	[0.86,0.91]	0.78	[0.74,0.82]	0.22*	[0.18,0.26]
Foreman or supervisor of other workers	0.45	[0.35,0.55]	0.57	[0.43,0.70]	0.79	[0.72,0.87]	0.88	[0.82,0.94]	0.86	[0.79,0.93]	0.18*	[0.11,0.25]
Skilled manual work	0.45	[0.40,0.50]	0.59	[0.52,0.65]	0.75	[0.71,0.79]	0.85	[0.82,0.88]	0.80	[0.76,0.84]	0.20**	[0.16,0.24]
Ref: Semi-skilled/unskilled manual work & never worked	0.47	[0.42,0.52]	0.52	[0.45,0.58]	0.72	[0.67,0.76]	0.85	[0.82,0.88]	0.80	[0.76,0.83]	0.28	[0.24,0.32]
Other	0.47	[0.40,0.53]	0.56	[0.47,0.65]	0.73	[0.67,0.78]	0.85	[0.81,0.90]	0.80	[0.75,0.85]	0.28	[0.22,0.34]
*N*	4428		2065		4428		4428		4428		4428	

Note. ^a^Yes = ‘trust them a lot/mostly’; No = ‘Distrust them a lot/mostly’ and ‘Don’t know’.

^b^Since the question about devolved government was only asked of participants in Scotland, Wales and Northern Ireland, Scotland (rather then England) is used as a reference category for these models.

* *p* < 0.05, ** *p* < 0.01, *** *p* < 0.001

In terms of sources of advice used, respondents in Scotland, Wales and Northern Ireland were less likely to use the UK government website for advice (PP 35%, 47% and 42%, respectively) compared to respondents in England (PP 54%) (Online Supplement Tables S3a and S3b). Use of devolved government website advice was highest in Scotland (PP 58%) and lower in Wales and Northern Ireland (PP 49% and 48%). Online Supplement Tables S3a and S3b also show some national differences in sources of advice used, although most differences were within 10 percentage points. For example, people in Scotland were most likely to use leaflets sent to them for advice (PP 20%) compared to people in Wales (PP 12%, calculated difference significant at *p* ≤ 0.001; not shown).

There was considerable variation by age in trust and use of some, but not all, sources of advice. Trust in the UK government ranged from a PP of 35% among the youngest age group to 62% among those aged 65 years or older. Older people were more likely to trust scientists and the NHS. Trust in family and friends followed a similar pattern. Older people were less likely to report using an NHS website, social media or messenger services for advice and more likely to use print newspapers, television and leaflets. There were particularly strong differences in predicted probabilities at the extremes of the age spectrum, such as for television (PP 33 percentage point difference) and social media (PP 26 percentage point difference). In contrast, we did not identify significant gender differences. Men were less likely to use the UK government, devolved government and NHS websites, social media and family and friends for advice, and more likely to report not using any sources, although differences in PPs were small (3–7 percentage points).

We found significant differences by annual household income for trust in the UK government, the NHS, and family and friends, with higher incomes correlating with greater likelihood of trust. Similar patterns were seen for use of advice from the UK government and an NHS website, but the overall evidence of social stratification by household income was not strong. Differences by occupational class were more pronounced. Those in higher professional groups and managers were more likely to report greater trust in devolved government, scientists and also religious leaders. Differences in occupational class were significant for all sources of information on COVID-19 used, except for television and social media. The greatest contrasts were found for devolved government sources between those in professional/higher technical work (PP 66%) and those in semi-skilled/unskilled manual workers and those who never worked (PP 45%).

### Vaccination status

Table 3 presents findings on trust in, and use of, different sources of advice and information on COVID-19 in relation to vaccination status.

**Table 3.** Vaccination status by trust and use of sources of COVID-19 pandemic advice (*N* = 4428).

Weighted analysis	Vaccinated or intending to get vaccinated			
	**OR**	**95% CI**	**PP**	**95% CI**
***Trust is sources of advice on COVID-19***				
**UK government**^a^	2.55***	[1.81,3.59]	0.97	[0.96,0.98]
*Ref: little to no trust*^a^			0.92	[0.91,0.94]
**Scottish government** (*N*:1160)	1.93*	[1.03,3.60]	0.97	[0.95,0.98]
*Ref: little to no trust*			0.94	[0.90,0.97]
**Welsh government** (*N*:670)	5.74***	[2.48,13.25]	0.98	[0.97,1.00]
*Ref: little to no trust*			0.91	[0.86,0.97]
**Northern Ireland government** (*N*:235)	5.43*	[1.19,24.69]	0.98	[0.94,1.02]
*Ref: little to no trust*			0.88	[0.79,0.98]
**The NHS**	3.44***	[2.45,4.83]	0.96	[0.95,0.97]
*Ref: little to no trust*			0.87	[0.83,0.90]
**Scientists**	3.28***	[2.35,4.57]	0.96	[0.95,0.97]
*Ref: little to no trust*			0.88	[0.86,0.91]
**Family and friends**	1.29	[0.92,1.81]	0.95	[0.94,0.96]
*Ref: little to no trust*			0.93	[0.91,0.95]
**Religious leaders**	1.24	[0.86,1.78]	0.94	[0.93,0.95]
*Ref: little to no trust*			0.95	[0.94,0.97]
***Sources used for information on COVID-19***				
**UK government website**	1.72***	[1.25,2.36]	0.96	[0.95,0.97]
*Ref: did not use source*			0.93	[0.92,0.94]
**Scottish government website** (*N*:1160)^b^	2.87***	[1.54,5.36]	0.97	[0.96,0.99]
*Ref: did not use source*			0.93	[0.90,0.96]
**Welsh government website** (*N*:670)^b^	1.11	[0.30,4.13]	0.97	[0.95,0.99]
*Ref: did not use source*			0.95	[0.91,0.98]
**Northern Ireland government website** (*N*:235)^b^	1.10	[0.30,4.11]	0.92	[0.85,0.99]
*Ref: did not use source*			0.91	[0.82,1.00]
**Local government website**	1.08	[0.74,1.58]	0.95	[0.93,0.96]
*Ref: did not use source*			0.94	[0.93,0.95]
**NHS Website**^c^	2.14***	[1.57,2.92]	0.96	[0.95,0.97]
*Ref: did not use source*			0.92	[0.91,0.94]
**Your GP (General Practitioner)**	1.68*	[1.04,2.74]	0.96	[0.95,0.98]
*Ref: did not use source*			0.94	[0.93,0.95]
**Leaflets/information brochures sent to you**	1.26	[0.80,1.99]	0.94	[0.93,0.95]
*Ref: did not use source*			0.95	[0.94,0.97]
**Television**	1.45*	[1.03,2.05]	0.93	[0.92,0.95]
*Ref: did not use source*			0.95	[0.94,0.97]
**Print newspaper**	1.40	[0.86,2.26]	0.96	[0.94,0.98]
*Ref: did not use source*			0.94	[0.93,0.95]
**Website of a newspaper**	1.28	[0.84,1.93]	0.95	[0.94,0.97]
*Ref: did not use source*			0.94	[0.93,0.95]
**Radio**	1.20	[0.80,1.80]	0.95	[0.93,0.97]
*Ref: did not use source*			0.94	[0.93,0.95]
**Social media**	0.56***	[0.40,0.79]	0.92	[0.89,0.94]
*Ref: did not use source*			0.95	[0.94,0.96]
**Messenger services** (e.g. Whatsapp)	0.72	[0.39,1.32]	0.93	[0.88,0.97]
*Ref: did not use source*			0.95	[0.94,0.95]
**Friends and family**	0.65**	[0.48,0.90]	0.93	[0.91,0.95]
*Ref: did not use source*			0.95	[0.94,0.96]
**None of the above**	0.44***	[0.30,0.66]	0.87	[0.83,0.91]
*Ref: did not use source*			0.94	[0.93,0.95]

Note. Logit models control for household income, occupational class, age, gender, UK nation.

^a^Yes = ‘trust them a lot/mostly’, No = ‘Distrust them a lot/mostly’ and ‘Don’t know’.

^b^Model run on sub-sample for each nation separately.

^c^Can refer to NHS England (https://www.nhs.uk/) or NHS websites in the devolved nations (e.g. Scotland: https://www.nhsinform.scot/).

* *p* < 0.05, ** *p* < 0.01, *** *p* < 0.001

Within-nation models looking at trust in devolved governments indicated that trust in advice from the Welsh and Northern Ireland government was associated with more than five-fold odds of being vaccinated or intending to get vaccinated. Trust in UK and Scottish government were also associated with a two-fold or higher chance of a positive vaccination status. The largest odds for positive vaccination status were found for trust in the NHS (OR 3.44) and trust in scientists (OR 3.28). In terms of predicted probabilities, 87% of those who did not trust the NHS reported a positive vaccination status, compared to 96% of those who did trust the NHS.

Use of UK and Scottish government (Scotland only) websites were both associated with a positive vaccination status. Use of an NHS website, the GP, and television were also associated with positive vaccination status, while use of social media, family and friends, and ‘none’ of the sources had a lower odds of being or intending to get vaccinated. Differences in PPs were small but statistically significant.

Table 4 shows vaccination status by sociodemographic characteristics, controlling for general distrust across sources. The 20% of the respondents who were least likely to trust different sources of information on COVID-19 were more likely to be vaccine-hesitant. There were no statistically significant differences between UK nations or genders. Respondents aged 65 and over had a four-fold greater odds of a positive vaccination status compared to the youngest group, and respondents least likely to be vaccinated were people aged 25–34 years (PP 90%). Overall proportions of being vaccinated or planning to get the vaccine were high. There was a small income gradient in vaccination status, with lower income groups slightly more vaccine-hesitant. There was some stratification by occupational class, but the patterns were not consistent. We found no significant interaction effects for vaccination status between: devolved nation and occupational class; devolved nation and income; general trust and age; gender and occupational class; or gender and age.

**Table 4.** Vaccination status by level of distrust and sociodemographic characteristics.

Weighted logit models	Vaccinated or intending to get vaccinated			
	**OR**	**95% CI**	**PP**	**95% CI**
**Distrust**				
High distrust (top 20%)	0.34***	[0.24,0.46]	0.89***	[0.86,0.91]
Ref: Lower distrust	1.00	[1.00,1.00]	0.96	[0.95,0.97]
**Nation**				
Ref: England	1.00	[1.00,1.00]	0.95	[0.94,0.96]
Scotland	1.05	[0.74,1.50]	0.95	[0.94,0.97]
Wales	1.20	[0.79,1.82]	0.96	[0.94,0.97]
Northern Ireland	0.83	[0.47,1.48]	0.94	[0.91,0.97]
**Gender**				
Female	0.97	[0.69,1.36]	0.95	[0.94,0.96]
Ref: Male	1.00	[1.00,1.00]	0.95	[0.94,0.96]
**Age**				
Ref: 18–24	1.00	[1.00,1.00]	0.93	[0.90,0.96]
25–34	0.71	[0.41,1.24]	0.90*	[0.87,0.93]
35–44	0.91	[0.52,1.62]	0.92	[0.90,0.95]
45–54	1.26	[0.71,2.24]	0.94	[0.92,0.96]
55–64	1.56	[0.87,2.81]	0.95	[0.94,0.97]
65 or older	3.97***	[1.94,8.13]	0.98**	[0.97,0.99]
**Annual household income**				
Ref: Under £19,999	1.00	[1.00,1.00]	0.92	[0.90,0.94]
£20,000–£39,999	1.54*	[1.05,2.26]	0.95*	[0.93,0.96]
3. £40,000–£59,999	2.07**	[1.19,3.60]	0.96**	[0.94,0.98]
4.£60,000–£79,999	2.95**	[1.35,6.41]	0.97***	[0.95,0.99]
5.£80,000–£99,999	0.91	[0.34,2.45]	0.92	[0.84,0.99]
6.£100,000–£119,999	2.29	[0.74,7.07]	0.97	[0.93,1.00]
Don’t know, prefer not to say	2.23**	[1.30,3.81]	0.96***	[0.95,0.98]
**Occupational class**				
Professional or higher technical work	1.45	[0.82,2.59]	0.95	[0.92,0.97]
Manager or Senior administrator	1.89^*^	[1.09,3.27]	0.96^*^	[0.94,0.98]
Junior manager	1.72	[0.92,3.20]	0.95	[0.93,0.98]
Non-managerial, non-manual work	2.27**	[1.31,3.92]	0.96**	[0.95,0.98]
Foreman or supervisor of other workers	1.95	[0.74,5.16]	0.96	[0.92,1.00]
Skilled manual work	1.22	[0.76,1.94]	0.94	[0.91,0.96]
Ref: Semi-skilled/unskilled manual work & never worked	1.00	[1.00,1.00]	0.92	[0.90,0.95]
Other	1.99*	[1.12,3.56]	0.96**	[0.94,0.98]
***N***	4428			

Note. Exponentiated coefficients; 95% confidence intervals in brackets.

*p < 0.05, **p < 0.01, ***p < 0.001

## Discussion

This study explored how the use of, and trust in, different sources of advice and information on COVID-19 differed between UK nations and across sociodemographic characteristics, and whether such patterns predicted COVID-19 vaccination status. Overall, we found high levels of trust in the key sources of public health advice and there was a positive association between using official sources of advice and vaccination intentions, even in the context of overall high vaccination rates. Levels of trust were highest for the NHS and for scientists and this did not differ by key sociodemographic characteristics. This finding contrasts with general public views on the NHS, with a 2021 survey showing that over half of respondents (57%) found that the general standard of care provided by the NHS had worsened during the preceding years.^15^ Interpretation of our findings has to be set in the context of the wider pandemic: at the time of data collection in summer 2021, the UK had emerged from exceptionally high levels of hospitalisations linked to COVID-19; yet, the vaccine roll-out was generally perceived as a UK success story.^16^ Overall, our findings highlight the need for the UK and devolved governments to value the importance of public trust in the health system and take appropriate measures to avoid undermining such trust.

The discrepancy between low trust in government and high trust in health professionals deserves some attention. One possible explanation for this could be the NHS’s perceived operational independence from governments and political figures, which might explain why trust in the NHS remained very high, even when trust in government was low. Research on the cultural history behind the British affection and attachment to the NHS as an institution may also go some way in explaining the high levels of trust in the NHS, and this provides relevant context here for international audiences.^17^ A different study design would however be required to explore this hypothesis.

The comparatively rarer mention of using GPs as a source of information might be explained by the availability of other trusted NHS sources (NHS websites, leaflets), which were also likely easier to access. Also, especially at the beginning of the pandemic, the number of consultations with primary care practices dropped substantially, although by September 2020, the total number of consultations had recovered to prepandemic levels.^18^

Our data suggest that respondents in Scotland and Wales had higher levels of trust in their respective governments than they had in the central UK government. This might simply reflect that people seek out the most relevant, rather than the most trusted, advice since there were differences in how the devolved nations implemented restrictions and vaccination roll-outs. However, other work points to higher levels of trust in Scotland prepandemic^19^ and the observed differences in our study may at least in part reflect longer term differences in trust in the devolved nations. This may have been further impacted by UK politics during the pandemic period, with a series of scandals including apparent breaches of COVID-19 regulations by senior members of the UK government, sometimes referred to as the ‘Cummings effect’ (after Dominic Cummings, senior aide to the UK prime minister at the time),^13^ and this may have further undermined public trust in the central UK government.

We found that religious leaders were generally less trusted as a source of advice and information on COVID-19 except for respondents in Northern Ireland. This observation is likely to reflect higher levels of religious affiliation in Northern Ireland compared to the rest of the UK. We did not collect data on religious affiliation but according to 2021 census data, only 17.4% of the Northern Ireland population reported no religious affiliation compared to 48% in Scotland.^20,21^ Trust in religious leaders was not associated with higher vaccination rates however. Evidence from Scotland suggests that faith leaders could be effectively engaged in health promotion initiatives,^22^ as is done in other parts of the world.^23^ There is scope for working with religious leaders to promote accurate health information and to address misinformation, which could contribute to higher vaccination uptake among population groups who rely on such leaders for advice.^24^

Social media and messenger apps were more frequently mentioned as sources of advice and information among young adults, and this was associated with lower probability of vaccination. We do not know what types of social media sources individuals used, but our findings align with other evidence that social media platforms can help spread misinformation, increase levels of distrust in official sources of advice,^25,26^ and this may have contributed to vaccine hesitancy during the pandemic.^6^ This raises important points about the need to address misinformation on social media using appropriate regulation, and the need to harness digital platforms to deliver targeted public health messaging tailored to different audiences.

We did not find pronounced variation by sociodemographic characteristics in relation to trust in the NHS. However, we found older age to be important in predicting trust in the UK government as well as the use of print newspapers and television, while younger respondents were more likely to use social media and messenger services as noted above. This observation points to a possible ‘generational divide’ in how different age groups engage with these information sources more broadly.^27^ Occupational class differences were evident for most sources explored, and occupational class differences were in part pronounced in the use of devolved government advice, and to some extent in the use of no sources.

### Limitations

Surveys such as that used in this study rely on self-reported data on issues that may be considered sensitive, such as household income and occupational class, with the former showing a high number of missing values. As noted above, we did not ask about religious affiliation, which might have helped explain findings on trust in religious leaders. Our survey sample also includes a higher percentage of people reporting to have been or intending to get vaccinated (93%) than what is known from official data (just under 80% at the end of 2021^1^). Plausible explanations are social desirability in survey responses and/or bias due to oversampling of some population groups, which the survey weights have not completely corrected for. The ‘vaccine-sceptical’ subsample (7.1%) was highly selective, and subsample characteristics may be further affected by differential COVID-19 survival rates for the vaccinated versus unvaccinated. Further, the survey was conducted using an online panel, which means that people who do not engage with the internet and digital means will be underrepresented among respondents. While we cannot be certain about the degree of bias introduced by the latter, it is likely that it has led to an overestimation of the proportion of people using digital sources of advice. Finally, our data capture people’s perceptions and intentions at a particular point during the COVID-19 pandemic and it may well be that survey responses would be different had the survey been fielded at an earlier or later period during the rapidly changing pandemic context.

## Supplemental Material

Supplemental Material - Trust in COVID-19 information sources and vaccination status: Exploring social inequalities and differences within the four United Kingdom nations using a representative survey Supplemental Material for Trust in COVID-19 information sources and vaccination status: Exploring social inequalities and differences within the four United Kingdom nations using a representative survey by Valeria Skafida, and Elke Heins in Journal of Health Services Research & Policy
