# Supplementary material for: Trust in COVID-19 information sources and vaccination status: Exploring social inequalities and differences within the four United Kingdom nations using a representative survey
Source: J Health Serv Res Policy. 2024 Feb 5;29(3):153–62. doi: 10.1177/13558196241227749 (PMC11196867; doi:10.1177/13558196241227749)
Supplement: Supplemental Material - Trust in COVID-19 information sources and vaccination status: Exploring social inequalities and differences within the four United Kingdom nations using a representative survey [file sj-pdf-1-hsr-10.1177_13558196241227749.pdf]

Online Supplement

Survey design

Data collection for our survey took place between 16-31 July 2021. In total 4428 adults (18+ years old) who resided in the UK took part. The survey company Bilendi (https://www.bilendi.co.uk/) uses online panels for data collection (2.5 million registered panellists) and invitations to participate in the survey were staggered and adjusted by continuously monitoring the achieved responses in relation to our set quotas. To obtain representative samples large enough for multivariate statistical analysis, the survey design over-sampled for the three smaller UK nations, Scotland, Wales, Northern Ireland.

Survey quotas intended to match the achieved sample to the UK population using key characteristics: gender, age, geographical region, and household socio-occupational class. To sample a varied profile of participants across constituent units in the UK, key characteristics quotas were implemented within regions in England and within council area groups in the other UK nations. Survey weights were used for all analyses and these adjusted the sample to known population proportions in the UK.

Table S1 shows the difference between the sample characteristics (unweighted), and weighted descriptive statistics which correct for accurate UK population ratios. Only 53% of sample participants were from England, but after application of survey weights, they represented 84% of the total UK population. The Northern Ireland sample was smaller than desired, potentially affecting inferences that can be made from this sub-sample to the Northern Ireland population.

Table S1 further presents data on weighted and unweighted statistics for the demographic variables which we controlled for in our analyses. This shows that, compared to the characteristics of the UK population, we oversampled participants in higher paid occupations, and slightly oversampled women. We also slightly under-sampled younger participants and oversampled those in the 55-64 age range.

Table S1. Weighted sample characteristics (N = 4428)

DEMOGRAPHICS	Weighted %	Unweighted sample size	Unweighted %
**Gender**			
Female	51.1	2490	56.2
Male	48.9	1927	43.5
Other	0	11	0.2
**Age**			
18-24	10.6	345	7.8
25-34	17	540	12.2
35-44	16	749	16.9
45-54	16.9	794	17.9
55-64	15.9	910	20.6
65 or older	23.6	1090	24.6
**Annual household income**			
Under £19,999	27.9	1135	25.6
£20,000 - £39,999	33.8	1470	33.2
3. £40,000 - £59,999	14.6	721	16.3
4. £60,000 - £79,999	6.6	325	7.3
5. £80,000 - £99,999	2.7	141	3.2
6. £100,000 -£119,999	2.8	135	3
Don't know, prefer not to say	11.7	501	11.3
**Nation**			
England	84.3	2363	53.4
Scotland	8.1	1160	26.2
Wales	4.7	670	15.1
Northern Ireland	2.8	235	5.3
**Occupational class**			
Professional or higher technical work	9.7	656	14.8
Manager or Senior Administrator	12.5	839	18.9
Junior Manager	12.3	446	10.1
Non-managerial, non-manual work	18.5	641	14.5
Foreman or Supervisor of Other Workers	3.9	149	3.4
Skilled Manual Work	17	646	14.6
Semi-Skilled/Unskilled Manual Work & never worked	18.1	704	15.9
Other	8.0	347	7.8

Table S.2 Vaccination status by trust and use of sources of advice and information on Covid-19 (N=4428)

	England (N=2363)		Scotland (N=1160)		Wales (N=670)		Northern Ireland (N=235)	
Weighted analysis[b]	PP	95%CI	OR	95%CI	PP	95%CI	PP	95%CI
Trust is sources of advice on Covid-19								
**UK Government**[a]	0.97	[0.96,0.98]	0.97	[0.96,0.98]	0.97	[0.96,0.99]	0.96	[0.94,0.98]
Ref: little to no trust[a]	0.92	[0.91,0.94]	0.93	[0.91,0.95]	0.93	[0.91,0.96]	0.91	[0.86,0.96]
**Devolved Government**	n/a		0.98	[0.97,1.00]	0.98	[0.94,1.02]	0.97	[0.95,0.98]
Ref: little to no trust	n/a		0.91	[0.86,0.97]	0.88	[0.79,0.98]	0.94	[0.90,0.97]
**NHS**	0.96	[0.95,0.97]	0.96	[0.95,0.97]	0.96	[0.95,0.98]	0.95	[0.92,0.98]
Ref: little to no trust	0.87	[0.83,0.90]	0.87	[0.82,0.92]	0.89	[0.84,0.93]	0.84	[0.76,0.92]
**Scientists**	0.96	[0.95,0.97]	0.96	[0.95,0.97]	0.97	[0.95,0.98]	0.95	[0.93,0.98]
Ref: little to no trust	0.88	[0.85,0.91]	0.88	[0.84,0.92]	0.90	[0.85,0.94]	0.87	[0.79,0.94]
**Family and friends**	0.95	[0.94,0.96]	0.95	[0.93,0.96]	0.95	[0.94,0.97]	0.94	[0.90,0.97]
Ref: little to no trust	0.93	[0.91,0.95]	0.94	[0.91,0.96]	0.94	[0.92,0.97]	0.92	[0.87,0.96]
**Religious leaders**	0.95	[0.94,0.97]	0.96	[0.94,0.97]	0.96	[0.94,0.98]	0.94	[0.91,0.97]
Ref: little to no trust	0.94	[0.93,0.95]	0.95	[0.93,0.96]	0.95	[0.93,0.97]	0.93	[0.89,0.97]
Sources used for information on Covid-19								
**UK Government website**	0.96	[0.95,0.97]	0.96	[0.95,0.98]	0.96	[0.95,0.98]	0.95	[0.92,0.98]
Ref: did not use source	0.93	[0.91,0.95]	0.94	[0.92,0.96]	0.94	[0.92,0.96]	0.92	[0.87,0.96]
**Devolved Government website**	n/a		0.97	[0.96,0.99]	0.97	[0.95,0.99]	0.92	[0.85,0.99]
Ref: did not use source	n/a		0.93	[0.90,0.96]	0.95	[0.91,0.98]	0.91	[0.82,1.00]
**'Local' government website**	0.95	[0.93,0.97]	0.95	[0.93,0.97]	0.96	[0.93,0.98]	0.94	[0.90,0.97]
Ref: did not use source	0.94	[0.93,0.96]	0.95	[0.93,0.96]	0.95	[0.93,0.97]	0.93	[0.89,0.97]
**NHS Website**	0.96	[0.95,0.97]	0.96	[0.95,0.98]	0.97	[0.95,0.98]	0.95	[0.93,0.98]
Ref: did not use source	0.92	[0.90,0.94]	0.93	[0.90,0.95]	0.93	[0.91,0.96]	0.91	[0.86,0.96]
**Your GP**	0.96	[0.95,0.98]	0.97	[0.95,0.98]	0.97	[0.95,0.99]	0.96	[0.92,0.99]
Ref: did not use source	0.94	[0.93,0.95]	0.95	[0.93,0.96]	0.95	[0.93,0.97]	0.93	[0.89,0.96]
**Leaflets/information brochures sent to you**	0.95	[0.93,0.97]	0.96	[0.94,0.98]	0.96	[0.94,0.98]	0.94	[0.91,0.98]
Ref: did not use source	0.94	[0.93,0.95]	0.95	[0.93,0.96]	0.95	[0.93,0.97]	0.93	[0.89,0.97]
**Television**	0.95	[0.94,0.97]	0.96	[0.94,0.97]	0.96	[0.94,0.98]	0.92	[0.88,0.96]
Ref: did not use source	0.93	[0.92,0.95]	0.94	[0.92,0.96]	0.95	[0.92,0.97]	0.92	[0.88,0.96]
**Print Newspaper**	0.96	[0.94,0.98]	0.96	[0.94,0.98]	0.96	[0.94,0.98]	0.95	[0.91,0.98]
Ref: did not use source	0.94	[0.93,0.95]	0.95	[0.93,0.96]	0.95	[0.93,0.97]	0.93	[0.89,0.97]
**Website of a newspaper**	0.95	[0.94,0.97]	0.96	[0.94,0.98]	0.96	[0.94,0.98]	0.94	[0.91,0.98]
Ref: did not use source	0.94	[0.93,0.95]	0.95	[0.93,0.96]	0.95	[0.93,0.97]	0.93	[0.89,0.96]
**Radio**	0.95	[0.93,0.97]	0.95	[0.93,0.98]	0.96	[0.94,0.98]	0.94	[0.90,0.98]
Ref: did not use source	0.94	[0.93,0.95]	0.95	[0.93,0.96]	0.95	[0.93,0.97]	0.93	[0.89,0.97]
**Social media**	0.92	[0.89,0.94]	0.92	[0.89,0.95]	0.93	[0.90,0.96]	0.90	[0.84,0.95]
Ref: did not use source	0.95	[0.94,0.96]	0.95	[0.94,0.97]	0.96	[0.94,0.97]	0.94	[0.91,0.97]
**Messenger services** (e.g. Whatsapp)	0.93	[0.88,0.97]	0.93	[0.88,0.97]	0.94	[0.89,0.98]	0.91	[0.84,0.98]
Ref: did not use source	0.95	[0.93,0.96]	0.95	[0.93,0.96]	0.95	[0.94,0.97]	0.93	[0.90,0.97]
**Friends and family**	0.93	[0.91,0.95]	0.93	[0.91,0.96]	0.94	[0.91,0.96]	0.91	[0.86,0.96]
Ref: did not use source	0.95	[0.94,0.96]	0.95	[0.94,0.97]	0.96	[0.94,0.97]	0.94	[0.91,0.97]
**None of the above**	0.87	[0.83,0.91]	0.87	[0.83,0.92]	0.89	[0.84,0.94]	0.84	[0.76,0.93]
Ref: did not use source	0.94	[0.93,0.95]	0.95	[0.93,0.96]	0.95	[0.93,0.97]	0.93	[0.90,0.97]

a. Yes = trust them a lot/mostly. No = Distrust them a lot/mostly & Don't know.
b. Logit models control for Household Income, Occupation Class, Age, Gender, UK Nation.
c. Model run on sub-sample for each nation separately.

Table S3a. Sociodemographic differences in use of different sources of information on Covid-19 (predicted probabilities)

	UK Government website		Devolved government[a]		Devolved government website		NHS website		Your GP		Print newspapers		Newspaper websites	
	**PP**	**95%CI**	**PP**	**95%CI**	**PP**	**95%CI**	**PP**	**95%CI**	**PP**	**95%CI**	**PP**	**95%CI**	**PP**	**95%CI**
**Nation**														
Ref: England	0.54	[0.52,0.56]			0.21	[0.20,0.23]	0.57	[0.55,0.59]	0.17	[0.15,0.18]	0.18	[0.16,0.19]	0.19	[0.18,0.21]
Ref2: Scotland	0.35***	[0.31,0.38]	0.58	[0.55,0.61]	0.17**	[0.14,0.19]	0.54	[0.51,0.58]	0.12***	[0.10,0.14]	0.15*	[0.12,0.17]	0.19	[0.16,0.21]
Wales	0.47*	[0.43,0.52]	0.49***	[0.44,0.53]	0.18	[0.15,0.22]	0.53	[0.49,0.58]	0.15	[0.12,0.18]	0.13**	[0.10,0.16]	0.18	[0.15,0.22]
Northern Ireland	0.42**	[0.34,0.49]	0.48*	[0.40,0.55]	0.14**	[0.09,0.18]	0.48*	[0.41,0.56]	0.16	[0.11,0.22]	0.20	[0.14,0.26]	0.21	[0.15,0.28]
**Gender**														
Ref: Female	0.54	[0.52,0.57]	0.57	[0.53,0.60]	0.22	[0.20,0.24]	0.59	[0.57,0.62]	0.16	[0.14,0.18]	0.17	[0.15,0.19]	0.19	[0.17,0.21]
Male	0.49*	[0.46,0.52]	0.50**	[0.46,0.54]	0.19	[0.17,0.22]	0.53***	[0.50,0.56]	0.16	[0.14,0.18]	0.18	[0.15,0.20]	0.19	[0.17,0.22]
**Age**														
Ref: 18-24	0.56	[0.50,0.62]	0.52	[0.40,0.63]	0.22	[0.17,0.27]	0.67	[0.61,0.73]	0.20	[0.16,0.25]	0.11	[0.07,0.14]	0.21	[0.16,0.26]
25-34	0.51	[0.46,0.57]	0.50	[0.42,0.57]	0.23	[0.19,0.28]	0.58*	[0.53,0.63]	0.16	[0.12,0.20]	0.11	[0.07,0.14]	0.21	[0.16,0.25]
35-44	0.50	[0.45,0.54]	0.56	[0.50,0.62]	0.18	[0.14,0.22]	0.52***	[0.47,0.56]	0.11***	[0.08,0.14]	0.14	[0.10,0.17]	0.20	[0.16,0.23]
45-54	0.52	[0.47,0.56]	0.57	[0.51,0.62]	0.21	[0.17,0.24]	0.59*	[0.55,0.64]	0.15	[0.12,0.18]	0.15	[0.12,0.18]	0.17	[0.14,0.21]
55-64	0.53	[0.49,0.57]	0.56	[0.51,0.61]	0.21	[0.18,0.24]	0.56**	[0.52,0.60]	0.17	[0.14,0.20]	0.22***	[0.18,0.25]	0.20	[0.17,0.24]
65 or older	0.50	[0.46,0.54]	0.51	[0.46,0.57]	0.20	[0.17,0.23]	0.51***	[0.47,0.54]	0.20	[0.17,0.23]	0.32***	[0.28,0.35]	0.19	[0.16,0.21]
**Annual household income**														
Ref: Under £19,999	0.47	[0.43,0.51]	0.52	[0.47,0.57]	0.21	[0.17,0.24]	0.52	[0.48,0.56]	0.20	[0.17,0.23]	0.17	[0.14,0.20]	0.19	[0.16,0.22]
£20,000 - £39,999	0.53*	[0.50,0.57]	0.54	[0.50,0.59]	0.22	[0.20,0.25]	0.59**	[0.56,0.62]	0.17	[0.14,0.19]	0.17	[0.14,0.19]	0.18	[0.16,0.21]
3. £40,000 - £59,999	0.57**	[0.52,0.62]	0.53	[0.47,0.60]	0.19	[0.15,0.23]	0.62**	[0.57,0.67]	0.14*	[0.11,0.17]	0.18	[0.14,0.21]	0.21	[0.17,0.24]
4. £60,000 - £79,999	0.57*	[0.50,0.64]	0.53	[0.42,0.64]	0.25	[0.19,0.32]	0.56	[0.48,0.64]	0.16	[0.11,0.21]	0.22	[0.16,0.29]	0.25	[0.19,0.31]
5. £80,000 - £99,999	0.50	[0.39,0.61]	0.52	[0.35,0.69]	0.22	[0.13,0.30]	0.53	[0.42,0.64]	0.13	[0.07,0.19]	0.21	[0.12,0.29]	0.26	[0.16,0.35]
6. £100,000 -£119,999	0.59*	[0.48,0.69]	0.47	[0.29,0.66]	0.17	[0.09,0.25]	0.58	[0.47,0.68]	0.16	[0.08,0.24]	0.27	[0.17,0.36]	0.27	[0.18,0.36]
Don’t know/won’t say	0.48	[0.43,0.53]	0.57	[0.49,0.65]	0.16	[0.13,0.20]	0.52	[0.47,0.58]	0.12	[0.09,0.15]	0.14	[0.11,0.18]	0.18	[0.13,0.22]
**Occupational class**														
Professional/higher technical work	0.55***	[0.50,0.60]	0.66***	[0.60,0.72]	0.24**	[0.20,0.28]	0.62***	[0.57,0.67]	0.20**	[0.16,0.25]	0.20	[0.16,0.24]	0.26***	[0.21,0.30]
Manager/Senior Admin.	0.59***	[0.55,0.64]	0.63***	[0.57,0.69]	0.24***	[0.20,0.28]	0.62***	[0.58,0.67]	0.19**	[0.16,0.23]	0.21*	[0.18,0.25]	0.23***	[0.19,0.27]
Junior Manager	0.62***	[0.56,0.67]	0.58*	[0.50,0.65]	0.25**	[0.20,0.30]	0.62***	[0.56,0.67]	0.16	[0.12,0.20]	0.21*	[0.17,0.26]	0.23**	[0.19,0.28]
Non-managerial, non-manual work	0.55***	[0.50,0.59]	0.58**	[0.52,0.65]	0.21*	[0.17,0.25]	0.57***	[0.52,0.61]	0.15	[0.12,0.18]	0.16	[0.13,0.20]	0.25***	[0.21,0.29]
Foreman/Supervisor of other workers	0.46	[0.36,0.56]	0.46	[0.33,0.58]	0.23	[0.15,0.31]	0.59*	[0.49,0.68]	0.14	[0.07,0.21]	0.15	[0.09,0.22]	0.07**	[0.03,0.11]
Skilled Manual Work	0.46	[0.41,0.51]	0.50	[0.43,0.56]	0.17	[0.14,0.21]	0.54**	[0.50,0.59]	0.16	[0.13,0.20]	0.17	[0.13,0.21]	0.17	[0.14,0.21]
Ref: Semi-Skilled/Unskilled Manual & never worked	0.43	[0.39,0.48]	0.45	[0.38,0.51]	0.15	[0.12,0.19]	0.46	[0.41,0.50]	0.12	[0.10,0.15]	0.15	[0.12,0.18]	0.14	[0.11,0.18]
Other	0.49	[0.42,0.55]	0.45	[0.36,0.55]	0.23*	[0.18,0.29]	0.57**	[0.51,0.63]	0.23***	[0.17,0.28]	0.12	[0.08,0.16]	0.15	[0.11,0.20]
**N**	4428		2065		4428		4428		4428		4428		4428	

Table S3b. Sociodemographic differences in use of different sources of information on Covid-19 (predicted probabilities)

	Television		Radio		Social Media		Messenger services		Family and friends		Leaflets		None of these	
	**PP**	**95%CI**	**PP**	**95%CI**	**PP**	**95%CI**	**PP**	**95%CI**	**PP**	**95%CI**	**PP**	**95%CI**	**PP**	**95%CI**
**Nation**														
Ref: England	0.52	[0.49,0.54]			0.17	[0.16,0.19]	0.03	[0.03,0.04]	0.29	[0.27,0.31]	0.15	[0.14,0.17]	0.08	[0.07,0.09]
Ref2[b]: Scotland	0.45**	[0.42,0.49]	0.15	[0.13,0.18]	0.19	[0.16,0.22]	0.03	[0.02,0.04]	0.26	[0.23,0.29]	0.20**	[0.17,0.22]	0.07	[0.06,0.09]
Wales	0.43***	[0.39,0.48]	0.16	[0.13,0.19]	0.17	[0.14,0.21]	0.03	[0.01,0.04]	0.23**	[0.19,0.27]	0.12**	[0.09,0.15]	0.09	[0.06,0.11]
Northern Ireland	0.49	[0.42,0.57]	0.22*	[0.16,0.28]	0.19	[0.13,0.26]	0.05	[0.02,0.08]	0.23	[0.17,0.29]	0.15	[0.10,0.20]	0.08	[0.04,0.12]
**Gender**														
Ref: Female	0.51	[0.48,0.53]	0.16	[0.14,0.19]	0.19	[0.17,0.21]	0.03	[0.02,0.04]	0.31	[0.29,0.34]	0.17	[0.15,0.19]	0.06	[0.05,0.08]
Male	0.51	[0.48,0.54]	0.17	[0.14,0.20]	0.16*	[0.14,0.18]	0.03	[0.02,0.04]	0.25*	[0.23,0.28]	0.14	[0.12,0.16]	0.10**	[0.08,0.11]
**Age**														
Ref: 18-24	0.36	[0.30,0.42]	0.20	[0.11,0.29]	0.36	[0.30,0.42]	0.08	[0.05,0.11]	0.36	[0.30,0.42]	0.11	[0.07,0.14]	0.09	[0.06,0.12]
25-34	0.33	[0.28,0.38]	0.10*	[0.06,0.15]	0.27*	[0.23,0.32]	0.06	[0.03,0.09]	0.28*	[0.23,0.33]	0.10	[0.07,0.13]	0.11	[0.08,0.14]
35-44	0.40	[0.35,0.44]	0.16	[0.11,0.20]	0.20***	[0.17,0.24]	0.05	[0.03,0.07]	0.26**	[0.22,0.30]	0.13	[0.09,0.16]	0.13	[0.10,0.16]
45-54	0.55***	[0.50,0.59]	0.18	[0.13,0.22]	0.17***	[0.14,0.20]	0.03**	[0.02,0.04]	0.28*	[0.24,0.32]	0.16	[0.13,0.20]	0.08	[0.05,0.10]
55-64	0.59***	[0.55,0.63]	0.16	[0.13,0.20]	0.13***	[0.10,0.15]	0.02***	[0.01,0.03]	0.28*	[0.24,0.31]	0.19***	[0.16,0.23]	0.06	[0.04,0.08]
65 or older	0.69***	[0.66,0.73]	0.20	[0.16,0.24]	0.10***	[0.07,0.12]	0.02***	[0.01,0.02]	0.28*	[0.24,0.31]	0.24***	[0.21,0.27]	0.05*	[0.03,0.06]
**Annual household income**														
Ref: Under £19,999	0.48	[0.44,0.52]	0.16	[0.12,0.21]	0.18	[0.15,0.21]	0.05	[0.03,0.06]	0.31	[0.28,0.35]	0.16	[0.13,0.19]	0.10	[0.08,0.12]
£20,000 - £39,999	0.54*	[0.50,0.57]	0.16	[0.13,0.19]	0.17	[0.15,0.20]	0.03	[0.02,0.05]	0.28	[0.25,0.31]	0.16	[0.14,0.19]	0.07*	[0.05,0.09]
3. £40,000 - £59,999	0.52	[0.47,0.57]	0.16	[0.12,0.21]	0.18	[0.14,0.21]	0.02*	[0.01,0.04]	0.26	[0.22,0.31]	0.15	[0.12,0.19]	0.05***	[0.03,0.07]
4. £60,000 - £79,999	0.52	[0.44,0.60]	0.18	[0.11,0.25]	0.16	[0.10,0.22]	0.02	[0.01,0.04]	0.29	[0.22,0.36]	0.09**	[0.06,0.13]	0.05**	[0.02,0.08]
5. £80,000 - £99,999	0.52	[0.41,0.64]	0.18	[0.07,0.28]	0.19	[0.10,0.27]	0.03	[0.00,0.05]	0.29	[0.19,0.38]	0.15	[0.09,0.21]	0.05	[-0.00,0.10]
6. £100,000 -£119,999	0.44	[0.33,0.54]	0.22	[0.06,0.38]	0.13	[0.06,0.20]	0.02	[0.00,0.05]	0.23	[0.14,0.31]	0.15	[0.07,0.22]	0.07	[0.02,0.12]
Don’t know/won’t say	0.47	[0.41,0.53]	0.18	[0.12,0.24]	0.19	[0.15,0.23]	0.02	[0.01,0.04]	0.25*	[0.21,0.30]	0.18	[0.14,0.22]	0.15*	[0.11,0.19]
**Occupational class**														
Professional or higher technical work	0.55	[0.50,0.60]	0.26***	[0.21,0.32]	0.17	[0.13,0.20]	0.05	[0.03,0.07]	0.33*	[0.28,0.38]	0.20***	[0.16,0.25]	0.06**	[0.04,0.08]
Manager or Senior Administrator	0.53	[0.49,0.58]	0.24***	[0.19,0.29]	0.17	[0.14,0.21]	0.06*	[0.04,0.08]	0.28	[0.24,0.33]	0.19***	[0.15,0.22]	0.05***	[0.03,0.06]
Junior Manager	0.52	[0.46,0.58]	0.23**	[0.16,0.29]	0.17	[0.13,0.22]	0.02	[0.01,0.04]	0.31	[0.25,0.36]	0.18**	[0.14,0.23]	0.04***	[0.02,0.07]
Non-managerial, non-manual work	0.53	[0.48,0.58]	0.20*	[0.15,0.26]	0.19	[0.16,0.23]	0.02	[0.01,0.04]	0.29	[0.25,0.34]	0.18***	[0.14,0.22]	0.08*	[0.06,0.11]
Foreman or Supervisor of Other Workers	0.46	[0.36,0.56]	0.10	[0.02,0.17]	0.15	[0.08,0.21]	0.03	[0.00,0.07]	0.25	[0.16,0.33]	0.13	[0.07,0.19]	0.09	[0.03,0.14]
Skilled Manual Work	0.46	[0.41,0.51]	0.16	[0.12,0.21]	0.16	[0.12,0.19]	0.03	[0.01,0.05]	0.24	[0.20,0.29]	0.13	[0.10,0.16]	0.10	[0.07,0.13]
Ref: Semi-Skilled/Unskilled Manual Work & never worked	0.49	[0.44,0.54]	0.12	[0.08,0.16]	0.18	[0.15,0.21]	0.03	[0.02,0.05]	0.26	[0.22,0.30]	0.10	[0.08,0.13]	0.12	[0.09,0.15]
Other	0.50	[0.43,0.56]	0.08	[0.03,0.13]	0.20	[0.15,0.26]	0.04	[0.02,0.06]	0.32	[0.25,0.38]	0.18**	[0.13,0.22]	0.12	[0.08,0.17]
**N**	4428		2065		4428		4428		4428		4428			

Note (a) Yes = ‘trust them a lot/mostly’; No = ‘Distrust them a lot/mostly’ and ‘Don’t know’.

Note (b) Since the question about devolved government was only asked of participants in Scotland, Wales and Northern Ireland, Scotland (rather then England) is used as a reference category for these models.
